# Supplementary material for: BUSCO Update: Novel and Streamlined Workflows along with Broader and Deeper Phylogenetic Coverage for Scoring of Eukaryotic, Prokaryotic, and Viral Genomes
Source: Mol Biol Evol. 2021 Jul 28;38(10):4647–54. doi: 10.1093/molbev/msab199 (PMC8476166; doi:10.1093/molbev/msab199)
Supplement: msab199_Supplementary_Data [file msab199_supplementary_data.zip › Manni_et_al_2021_Supplementary.pdf]

Supplementary file for:

"BUSCO update: novel and streamlined workflows along with broader and deeper phylogenetic coverage for scoring of eukaryotic, prokaryotic, and viral genomes"

Mosè Manni*, Matthew R Berkeley*, Mathieu Seppey*, Felipe A Simão, and Evgeny M Zdobnov+

Department of Genetic Medicine and Development, University of Geneva, and Swiss Institute of Bioinformatics, Geneva, Switzerland

Table of contents

Supplementary notes **2**
- Novel pipelines summary 2
- Comparison of BUSCO_MetaEuk and BUSCO_Augustus workflows 4
- Dataset selection benchmark 4
- BUSCO-CheckM comparison 4
- Cross-domain matches 5
- Benchmark using simulated incomplete genomes and gene sets 5
- Note on the interpretation of results 6
- Distribution 6

References for supplementary notes **7**

Supplementary figures **8**

Supplementary tables **20**

Supplementary notes

Novel pipelines summary

There are two distinct elements to the BUSCO package, the open-source software and the datasets created based on OrthoDB orthologous groups (Kriventseva et al. 2019; Zdobnov et al. 2021). The datasets interface directly with the BUSCO software and different datasets' versions (e.g. odb9 and odb10 datasets) are bound to specific versions of the software. Odb10 datasets can be used with BUSCO v4 and v5, whereas the superseded odb9 datasets cannot be used with these versions. Each dataset contains a configuration file with parameters that dictate which pipeline should be used in a given BUSCO run and influence various parameters and cutoff thresholds used in the BUSCO pipeline. The BUSCO v5 software contains an upgraded and expanded pipeline compared to v3. The white box at the centre of supplementary figure 2 contains the core BUSCO pipeline. The grey highlighted region is the v3 pipeline. The following is a summary of the major changes made to the software after v3:

- *Prodigal gene prediction.* In BUSCO v4, a separate pipeline was introduced for genome assemblies assessed using the prokaryotic datasets. This pipeline uses Prodigal for gene prediction (Hyatt et al. 2010), as it yields superior results on prokaryotic clades than the BLAST/Augustus combination, and increases the efficiency of the pipeline. For runs using the mollicutes_odb10 dataset, and its sub-datasets mycoplasmatales_odb10 and entomoplasmatales_odb10, there is some ambiguity regarding the appropriate translation table to use during the gene prediction step, as some bacteria in these clades use a different genetic code then the common bacterial genetic code. To address this, the coding density of the input file is determined after Prodigal completes. If the coding density is below a threshold of 80%, Prodigal is run again, this time specifying the translation table 4 instead of 11. These parameters are controlled using the values found in the dataset configuration file. Whichever genetic code setting yields a predicted gene set with a higher coding density is selected as optimal. An exception to this arises when the coding densities for both runs are within 5%. In this case, BUSCO examines the scores assigned by Prodigal to the genes found. The mean scores assigned to each gene prediction are calculated and the run with the highest mean score is chosen.

- *BUSCO_MetaEuk workflow.* For v5, the MetaEuk (Levy Karin et al. 2020) gene predictor was introduced as the default for the eukaryota pipeline, replacing the BLAST/Augustus combination, though this pipeline is still available by passing the "--augustus" option. MetaEuk harnesses the fast and sensitive search capabilities of MMSeqs2 (Steinegger and Söding 2017), along with a dynamic programming procedure, to identify exons in an input assembly. The proteins used to construct the BUSCO datasets are used as reference sequences for MetaEuk. For a few datasets we increased the number of reference sequences by including BUSCO genes from additional species from OrthoDB10 to increase the sensitivity. The gene prediction step is run twice, the second time a new reference sequence database is created to include only BUSCO genes that were missing or fragmented after the first run. Some MetaEuk parameters are adjusted in the rerun to expand the parameters' space, e.g. in the initial run: min_exon_aa = 15; max_overlap = 15; min_intron = 5, whereas in the rerun: min_exon_aa = 5; max_overlap = 5; min_intron = 1. The max_intron and max_seq_len parameters are specific to the dataset used and are set based on the parameters in the BUSCO dataset configuration.

- *Automated Lineage Selection*. For a BUSCO run, the mandatory user-provided arguments are the input file, the output directory name and the run mode (either "genome", "protein" or "transcriptome"). In v3, the lineage dataset to be used was also mandatory. V4 saw the introduction of the

"--auto-lineage" option, which can be used to automatically determine which dataset to use for the completeness assessment. The auto-lineage pipeline starts by running a complete BUSCO assessment using the three "root" datasets: archaea_odb10, bacteria_odb10 and eukaryota_odb10 (also see main text). The "--auto-lineage-prok" (archaea, bacteria) and "--auto-lineage-euk" (eukaryota) options provide further control, for when the user knows the assembly is definitely either prokaryotic or eukaryotic. The results of the "root" datasets are assessed and the highest scoring dataset is selected. According to the domain of origin, the single-copy marker genes identified in this first assessment are individually placed on one of three precomputed phylogenetic trees, using SEPP (Mirarab et al. 2011), which relies on pplacer (Matsen et al. 2010). Following the placements on the branches of the precomputed tree, if the marker genes were successfully placed, the selected node of the tree determines the BUSCO dataset to be used for a final run. To avoid dataset selection on the basis of possible spurious placements, BUSCO requires a minimal set of markers (12) to start the selection process. A minimal proportion of the total placements (0.55) is also required to select a lineage deeper in the taxonomy. These thresholds were empirically set by analyzing the outcome of the selection process when varying these parameters on available genomes. If the conditions for selecting a higher-resolution dataset are not met, BUSCO reverts to the results from the main "root" dataset corresponding to the predicted domain of the species. The precomputed trees for the phylogenetic placement for Bacteria, Archaea and Eukaryota were built with FastTree (Price et al. 2010) from a supermatrix of concatenated BUSCO markers extracted from a phylogenetically even subset of species present in OrthoDB v10. Since the compilation of BUSCO datasets follows the NCBI taxonomy (Schoch et al. 2020), a pruned multifurcating version of the NCBI taxonomic tree was used to constrain the topologies of the corresponding trees.

- *Virus pipeline.* V5 also saw the introduction of a virus assessment pipeline. There are 27 viral datasets currently available (supplementary table 1). These viral datasets can be specified as normal at the start of a BUSCO run. Alternatively, when using the "--auto-lineage" option, an automated virus detection pipeline is triggered if the result from all three "root" datasets (archaea, bacteria, eukaryota) contains less than 3% single-copy BUSCOs and the input file size is smaller than 500 KB, as current BUSCO datasets include viral species with genome sizes below this value. This pipeline uses the genome option for prokaryotes, i.e. Prodigal. Given the comparatively small size of viral sequences and datasets, it is possible to run the auto-lineage pipeline quickly, using all 27 of the virus datasets. The 27 resulting scores are assessed and the highest scoring dataset is returned.

- *Batch mode*. In v5, the user-provided input can now be a folder containing input files (genomes or gene sets). This is detected automatically and triggers the "batch mode". The output of this batch run is similar to normal, but each input file has a separate results folder contained within the main output directory. An additional summary file is created with the results of each input file available in tabular form.

- *Parasitic check*. The fungi_odb10 dataset contains a list of BUSCO marker genes that are commonly missing in Microsporidia, a sub-clade of fungi with a parasitic lifestyle and a reduced set of fungal marker genes. When 80% or more of these BUSCOs are reported as missing in the BUSCO results, and these missing BUSCOs themselves comprise at least 80% of all the missing BUSCOs reported, the marker genes on this list are removed from consideration and the scores are recalculated with the reduced total number of marker genes for the dataset. The user is clearly alerted when this is the case. While microsporidian_odb10 is the appropriate dataset for this species and should be used, this strategy tries to avoid massive underestimation of completeness when the more general fungi_odb10 dataset is used on these species. The same strategy may be introduced for other datasets/parasitic clades in the future.

Comparison of BUSCO_MetaEuk and BUSCO_Augustus workflows

The two workflows for eukaryotic assessment give comparable BUSCO estimates (see main text). However, the two gene predictors, using different approaches can have different performance in predicting the genes on the same species. We investigated if there were a consistent set of BUSCOs that score differently between the two prediction programs on a set of genomes. Supplementary figure 4 reports the number of differences in predictions on a set of 139 arthropod genomes using the arthropoda_odb10 dataset (1’013 markers). Overall, there were no consistent major differences in the ability to predict specific BUSCOs between the two workflows on this set of genomes, except for two BUSCOs (out of 1’013). OG (orthologous group) 66028at6656 was missed with higher frequency by BUSCO_Augustus (missing in 89 vs 3 species), while OG 165142at6656 was missed with higher frequency by BUSCO_Metaeuk (missing in 58 vs 12 species). No evident reason was found to account for such difference. In general the two workflows give similar estimation of missing genes, e.g. over 139 species the majority of BUSCOs fall between a -5 and +5 difference (supplementary fig. 4), with ~160 BUSCOs with no differences at all between the two workflows across all species. Overall there is a slight tendency for BUSCO_Metaeuk to find more BUSCOs (supplementary fig. 4). The differences observed between the two workflows is not surprising as the performance of different gene prediction methods is relative to the specific genome/gene under consideration, and the gene structures for the same BUSCOs can differ from species to species.

Dataset selection benchmark

436 bacterial and archaeal assemblies from RefSeq that are not part of the source used to construct the BUSCO datasets were randomly selected (supplementary table 7).
A constraint was applied to ensure that each BUSCO dataset was the most specific lineage to assess the completeness of up to five assemblies. For generic datasets (e.g. Bacteria), this means either organisms belonging to lineages not covered by BUSCO specific datasets, or genomes with incomplete taxonomic annotations, were selected. An annotation containing only the main seven taxonomic ranks was used, which excluded three datasets (bacteroidetes-chlorobi_group_odb10, rhizobium-agrobacterium_group_odb10, delta-epsilon-subdivisions_odb10), and six datasets could not be found as the most specific lineage for any of the assemblies sampled. BUSCO was run with the option “--auto-lineage-prok” and the choice of dataset was recorded. In one case, rhizobium-agrobacterium_group_odb10, which is not included in the seven “valid” benchmark annotations, was selected by BUSCO; in this case the selection was considered correct (as the species rightly belong to rhizobium-agrobacterium_group) and assigned to match the direct parent dataset, rhizobiales_odb10, which is supported by the benchmark annotations.
Choices made by BUSCO were categorized as follows: correct when the dataset selected is the most specific that matches the taxonomic annotation of the evaluated assembly; suboptimal when a dataset suitable for the analysis (i.e part of the lineage) but not the most specific was selected; and in disagreement with NCBI otherwise.

BUSCO-CheckM comparison

The same 436 genomes used for the dataset selection benchmark were used. The memory and runtimes were recorded using the Snakemake (Mölder et al. 2021) “benchmark” option. For memory consumption, “max_rss” values were used. BUSCO was run in batch mode using the “--auto-lineage-prok” option, while CheckM was run using its batch mode (lineage_wf) (fig. 3b, supplementary table 8). The runtime and memory required of both programs were recorded running with 30, 12 and 8 CPUs respectively, in a HPC environment (supplementary table 9 and 10). Running BUSCO through a workflow management system can considerably decrease the runtime on a set of prokaryotic genomes. Using Snakemake and allowing a total of 30 CPUs, with 5 CPUs for each

analysis, the runtime for assessing a set of 436 genomes decreases more than half (supplementary table 9).

Cross-domain matches

In the process of selecting the most specific dataset during the auto-lineage workflow, BUSCO automatically assesses the presence of markers of the bacteria_odb10, eukaryota_odb10 and archaea_odb10 datasets. These intermediate results are available to the users and are also reported as additional columns in the summary table when BUSCO is run in batch with auto-lineage. These results may be useful to spot contamination problems. However, due to cross-matches between datasets, a number of BUSCOs will likely be estimated also in the absence of contamination. We compiled the distribution and frequency of cross-domain BUSCOs (Archaea and Eukaryota) on 2'779 bacterial genomes from RefSeq (supplementary fig. 7, supplementary table 10); and of archaeal and bacterial BUSCOs on eukaryotes (fungal genomes and invertebrate gene sets from RefSeq) (supplementary fig. 8, supplementary table 10). Since genomes from RefSeq are of high quality and with few contaminations, these matches are likely due to cross-domain matches, thus not representing contamination signals. For the archaea_odb10 and eukaryota_odb10 datasets there is a high number of BUSCOs that are almost never found in bacterial species, and can represent good contamination signals (supplementary table 11). For archaea_odb10 and bacteria_odb10 in eukaryotic species the number of cross-matches is higher and their distribution tends to vary depending on the clade under consideration, e.g. pattern in fungi and invertebrates is different (supplementary fig. 8, supplementary table 11).

Benchmark using simulated incomplete genomes and gene sets

A Snakemake (v6.0.2) pipeline was set up to conduct this part of the benchmark. The BUSCO v5.1.2 Docker container maintained by our team was converted into a singularity container using the utilities quay.io/singularity/docker2singularity:v3.7.3 to be integrated in the Snakemake workflow and be run on our HPC environment. BUSCO v3.0.2 was also installed in a similar container. For each species of interest, the genomic file, the protein set, and the GFF file were retrieved. The evaluation follows two different approaches: A) randomly removing from the input files a predefined percentage of proteins and their corresponding genes across the whole annotated protein set, and B) randomly removing a predefined percentage of proteins and their corresponding genes in the subset that are predicted to correspond to BUSCO markers. This selection and matching between protein and genes were based on the GFF files and, in the case of approach B, on an additional mapping of the protein set to OrthoDBv10 to identify the BUSCO markers. The longest isoform of each protein was kept, with a random selection when confronted with multiple same-length candidates. Corresponding genes were masked according to their coordinates from the GFF using the bedtools v2.25.0 maskfasta utilities (Quinlan and Hall 2010). Each percentage of depletion was generated in five random replicates.

The lineages to be used for each species were defined manually for BUSCO v3 (odb9 sets) and BUSCO v5 (odb10 sets). For all input files generated with the approach A, BUSCO v5 was run in protein mode and genome mode, both with BUSCO_MetaEuk and BUSCO_Augustus workflows. BUSCO v3 was run in protein mode and genome mode. For all input files generated with the approach B, BUSCO v5 was run in protein mode and genome mode, both with BUSCO_MetaEuk and BUSCO_Augustus workflows.

The missing proportions of markers (M:xx%) were extracted from the BUSCO short_summary file and the results plotted with ggplot2 (Wickham 2009) (fig. 4, supplementary fig. 9-11). For approach B, the precision value [TP/(TP+FP)] is based on the prediction of all markers removed or kept from the input file, with true presence and true absence as TP, and false presence and false absence as FP. Markers that did not appear in the official protein set of the species of interest were ignored, as

their absence could be caused by either a true loss or an imperfect annotation of the genome that makes it impossible to define the ground truth. All the simulated data and intermediate results are available at https://www.doi.org/10.5281/zenodo.4972052.

Note on the interpretation of results

The primary goal of a BUSCO analysis is to provide an overall estimation of the completeness of genome assemblies and gene sets, especially for non-model organisms which usually lack extensive RNA-seq data or complete genomes from closely related species to estimate the quality of the assembly. A try and assess approach during genome assembly and annotation procedures enables the exploration of different methods and regions of parameter space until a plateau is reached in terms of BUSCO score. When applied in batch to MAGs, a BUSCO analysis will give an overview of the groups of organisms recovered in the metagenome, along with a sense of their completeness. The per-lineage and near-universal single copy orthologs approach is ideal for such an objective, as it enables the evaluation of any recovered genomes independently from having any genomic/taxonomic background of the organisms.

However, for the correct interpretation of the results, it is important to consider the limitations inherent to the BUSCO markers delineation. During evolution, within the same clade, gene duplications and losses occur, also in clades of closely related species. On top of that, currently available genomes/gene sets that are used to build the BUSCO datasets are rarely 100% complete and free of errors. Therefore, in each gene set, some genes may be absent or duplicated due to technical limitations and errors. To account for true variations and widespread incompleteness of gene sets, while being able to still recover a sufficient number of markers, we enforce a threshold of 90% (and not 100%) for the presence and single copy criteria necessary to retain a BUSCO marker.

Imprecisions down to single markers are possible, and may vary depending on the specific genome/dataset under consideration. Users manually curating gene predictions in relation to BUSCO markers should bear in mind the dynamics of gene evolution. True duplication could lead to real duplicated markers being reported, and users may focus on selecting the copy that has best retained the characteristic of the ancestral copy to be used in subsequent analyses. A gene annotated as a BUSCO marker might actually be the close paralog of a lost marker gene in that species. Some gene models may be easy to predict in some species and difficult in others, and true markers might be missed by the BUSCO genome mode while found in the protein set that was produced by a different annotation pipeline using ad-hoc species-specific evidence. Generally, a few imprecisions on large datasets, e.g. when recovering markers for phylogenomic analyses, should not have a large impact on the outcome, e.g. changing the topology of a phylogenomic tree. However, it is down to the user to judge if the level of precision offered by BUSCO is enough for the task at the end, keeping in mind that high-level curation of each gene is beyond the scope of the software itself.

Distribution

Our team maintains an up to date version of BUSCO on three distribution channels: Gitlab, Conda and Docker. The code repository can be cloned directly from Gitlab, however all third-party dependencies will need to be installed manually. The Conda package and Docker container are both ready to use out of the box. To facilitate a better integration with conda, the configuration file, which was mandatory for v3 and v4, has been relegated to an option ("--config") in v5, with the default behaviour now to use the dependency versions found in the environment path. Full installation and usage instructions are found in the online user guide: https://busco.ezlab.org/busco_userguide.html.

References for supplementary notes

Hyatt D, Chen G-L, LoCascio PF, Land ML, Larimer FW, Hauser LJ. 2010. Prodigal: prokaryotic gene recognition and translation initiation site identification. *BMC Bioinformatics* 11:119.

Kriventseva EV, Kuznetsov D, Tegenfeldt F, Manni M, Dias R, Simão FA, Zdobnov EM. 2019. OrthoDB v10: sampling the diversity of animal, plant, fungal, protist, bacterial and viral genomes for evolutionary and functional annotations of orthologs. *Nucleic Acids Research* 47:D807–D811.

Levy Karin E, Mirdita M, Söding J. 2020. MetaEuk—sensitive, high-throughput gene discovery, and annotation for large-scale eukaryotic metagenomics. *Microbiome* 8:48.

Matsen FA, Kodner RB, Armbrust EV. 2010. pplacer: linear time maximum-likelihood and Bayesian phylogenetic placement of sequences onto a fixed reference tree. *BMC Bioinformatics* 11:538.

Mirarab S, Nguyen N, Warnow T. 2011. SEPP: SATé-Enabled Phylogenetic Placement. In: Biocomputing 2012. Kohala Coast, Hawaii, USA: WORLD SCIENTIFIC. p. 247–258. Available from: http://www.worldscientific.com/doi/abs/10.1142/9789814366496_0024

Mölder F, Jablonski KP, Letcher B, Hall MB, Tomkins-Tinch CH, Sochat V, Forster J, Lee S, Twardziok SO, Kanitz A, et al. 2021. Sustainable data analysis with Snakemake. *F1000Res* 10:33.

Price MN, Dehal PS, Arkin AP. 2010. FastTree 2 – Approximately Maximum-Likelihood Trees for Large Alignments. *PLOS ONE* 5:e9490.

Quinlan AR, Hall IM. 2010. BEDTools: a flexible suite of utilities for comparing genomic features. *Bioinformatics* 26:841–842.

Schoch CL, Ciufo S, Domrachev M, Hotton CL, Kannan S, Khovanskaya R, Leipe D, Mcveigh R, O'Neill K, Robbertse B, et al. 2020. NCBI Taxonomy: a comprehensive update on curation, resources and tools. *Database* [Internet] 2020. Available from: https://doi.org/10.1093/database/baaa062

Steinegger M, Söding J. 2017. MMseqs2 enables sensitive protein sequence searching for the analysis of massive data sets. *Nature Biotechnology* 35:1026–1028.

Wickham H. 2009. ggplot2: Elegant Graphics for Data Analysis. New York: Springer-Verlag Available from: https://www.springer.com/de/book/9780387981413

Zdobnov EM, Kuznetsov D, Tegenfeldt F, Manni M, Berkeley M, Kriventseva EV. 2021. OrthoDB in 2020: evolutionary and functional annotations of orthologs. *Nucleic Acids Research* 49:D389–D393.

Supplementary figures

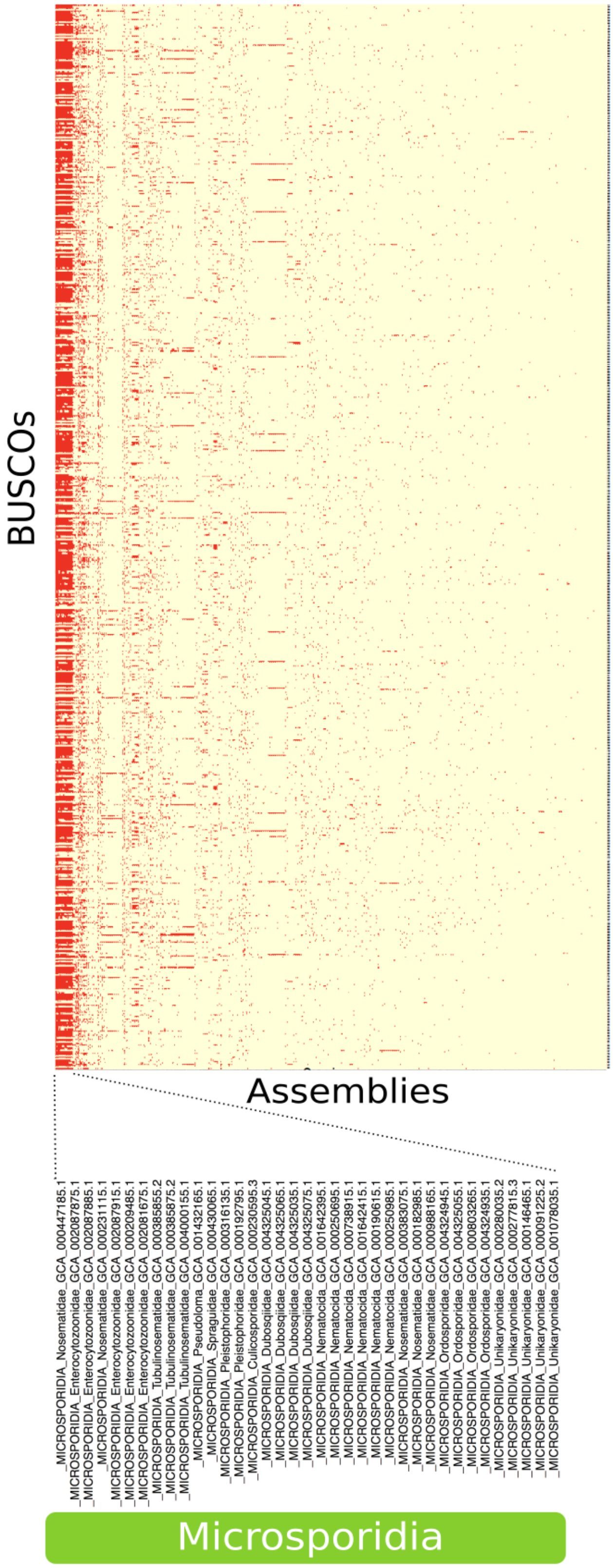

Supplementary Fig. 1. Presence (yellow)/absence (red) matrix of BUSCOs (rows) of the fungi_odb10 dataset (758 markers) in fungal gene sets (columns). Clades of microsporidian, on the left, lack on average a high proportion (~80%) of BUSCOs from the fungi_odb10 dataset. In general, as for all other clades, species are best assessed with the most specific and highest resolution dataset, in this case microsporidia_odb10 (see "microsporidia_odb10" panel in figure 2a).

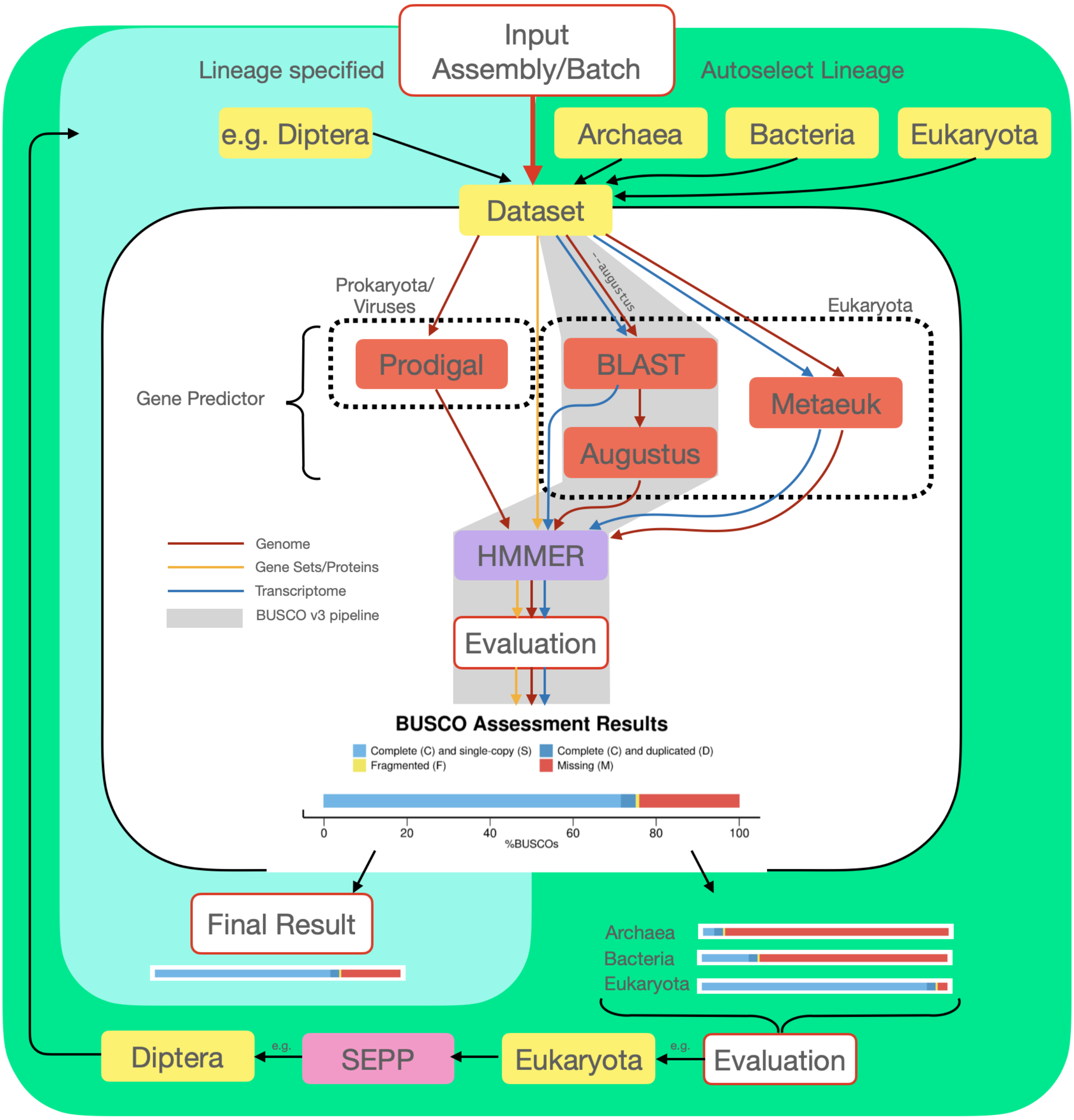

Supplementary Fig. 2. Overview of the BUSCO v5 pipeline. When running in automated lineage selection, the pipeline (white area) is run using the three "root" datasets, leading to a choice of more appropriate lineage specific dataset (green area). Then, or if the user provides a manual choice of dataset, the pipeline produces the final result (light blue area).

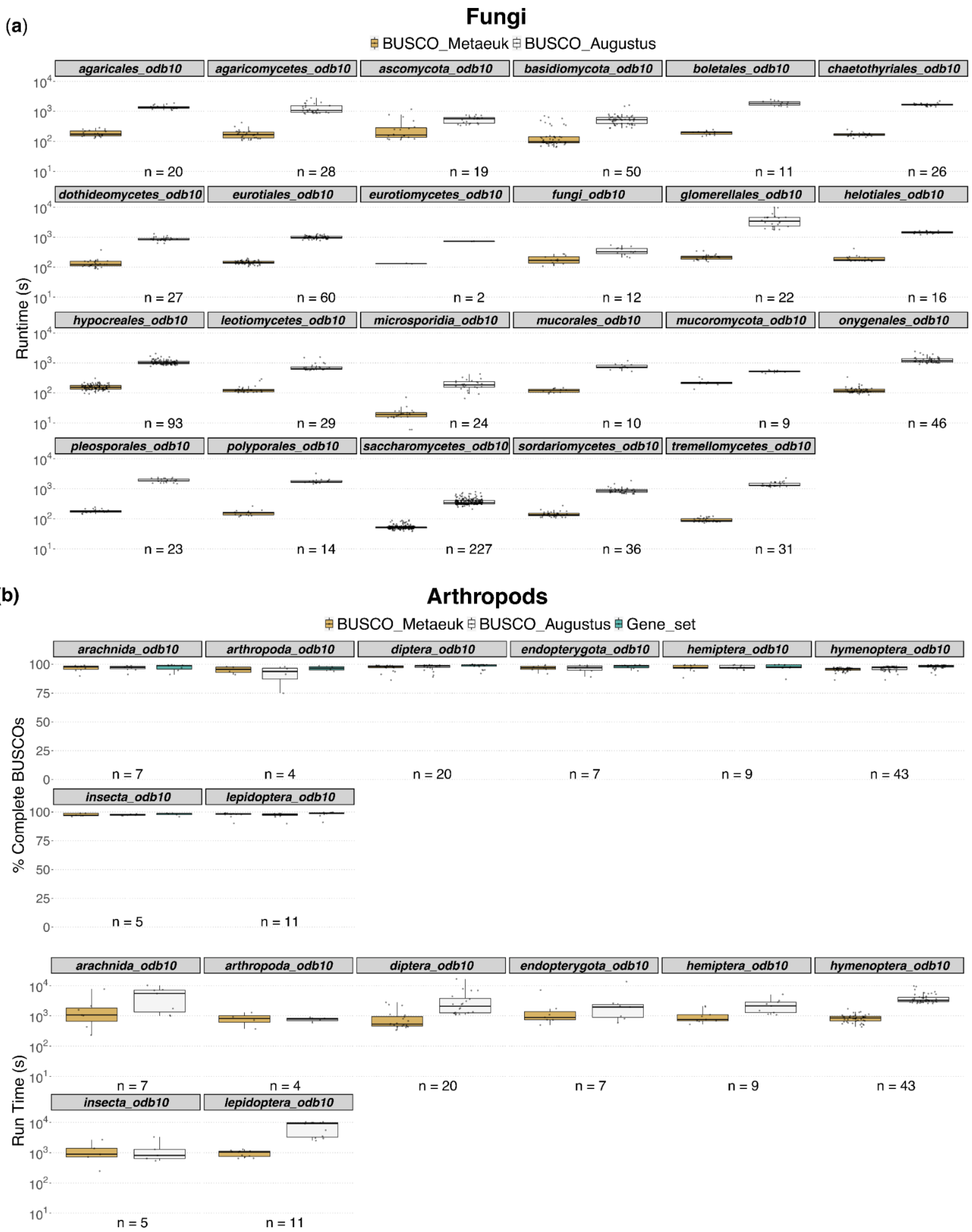
(a)
Fungi
BUSCO_Metaeuk BUSCO_Augustus
Runtime (s)
agaricales_odb10
n = 20
agaricomycetes_odb10
n = 28
ascomycota_odb10
n = 19
basidiomycota_odb10
n = 50
boletales_odb10
n = 11
chaetothyriales_odb10
n = 26
dothideomycetes_odb10
n = 27
eurotiales_odb10
n = 60
eurotiomycetes_odb10
n = 2
fungi_odb10
n = 12
glomerellales_odb10
n = 22
helotiales_odb10
n = 16
hypocreales_odb10
n = 93
leotiomycetes_odb10
n = 29
microsporidia_odb10
n = 24
mucorales_odb10
n = 10
mucoromycota_odb10
n = 9
onygenales_odb10
n = 46
pleosporales_odb10
n = 23
polyporales_odb10
n = 14
saccharomycetes_odb10
n = 227
sordariomycetes_odb10
n = 36
tremellomycetes_odb10
n = 31
(b)
Arthropods
BUSCO_Metaeuk BUSCO_Augustus Gene_set
% Complete BUSCOs
arachnida_odb10
n = 7
arthropoda_odb10
n = 4
diptera_odb10
n = 20
endopterygota_odb10
n = 7
hemiptera_odb10
n = 9
hymenoptera_odb10
n = 43
insecta_odb10
n = 5
lepidoptera_odb10
n = 11
Run Time (s)
arachnida_odb10
n = 7
arthropoda_odb10
n = 4
diptera_odb10
n = 20
endopterygota_odb10
n = 7
hemiptera_odb10
n = 9
hymenoptera_odb10
n = 43
insecta_odb10
n = 5
lepidoptera_odb10
n = 11

(c)

Protists

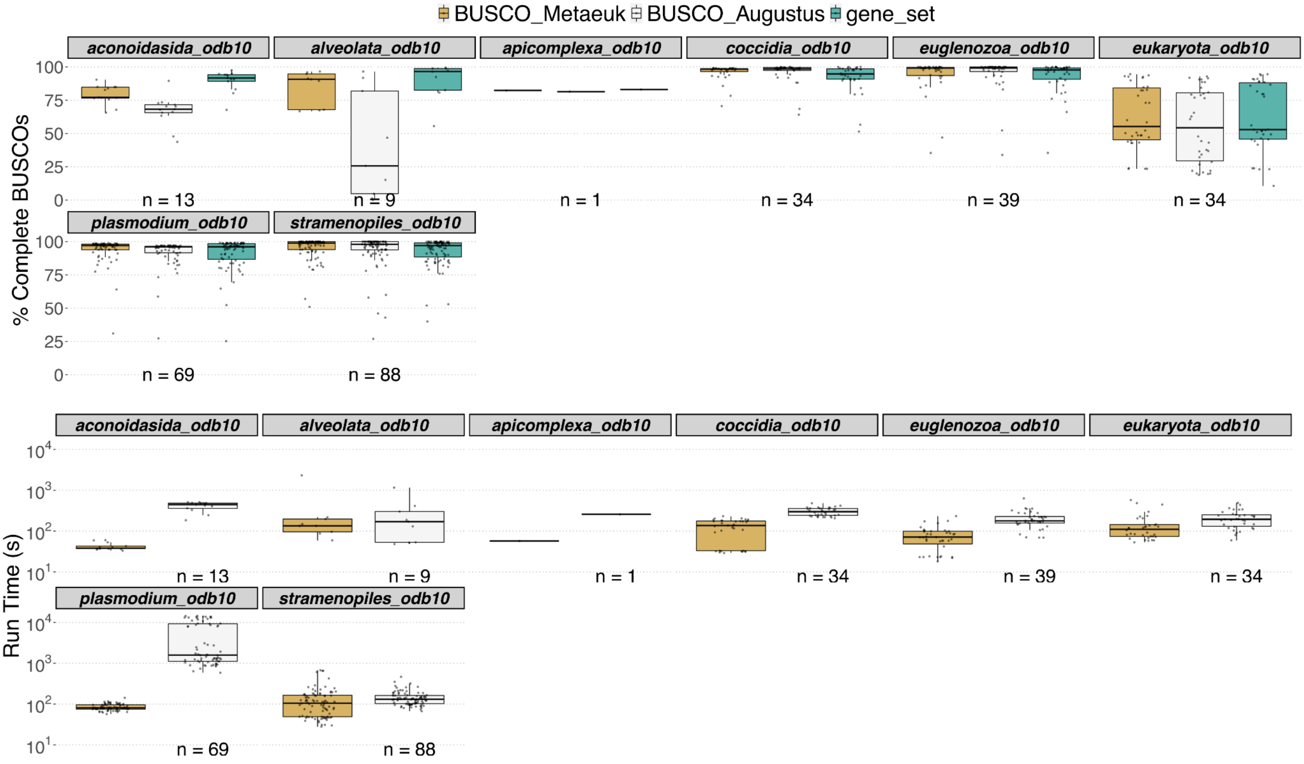

Supplementary Fig. 3. (a) Comparisons of BUSCO runtimes obtained using the two available workflows for eukaryotic species on the set of fungal genomes analyzed in figure 2a. The y-axis corresponding to runtimes is log-transformed. The newly-introduced BUSCO_MetaEuk workflow allows faster assessments. (b and c) Comparisons of BUSCO scores and runtimes obtained on a set of arthropod and protist genomes using the two available workflows for eukaryotic species. The upper panels display the percentage of complete BUSCOs for the BUSCO_MetaEuk (orange) and BUSCO_Augustus (white) workflows. Assessments on gene sets are also displayed for comparison (green). Genomes were assessed using the most specific available datasets, which is displayed at the top of each sub-panel. The bottom panels display the runtimes for BUSCO_MetaEuk (orange) and BUSCO_Augustus (white) workflows. The y axis (in seconds) of the runtime is log-transformed.

For these analyses we did not pass any extra parameters to BUSCO. Some protists, e.g. some species from the clade Alveolata, do not use the standard genetic code. In the “alveolata_odb10” boxplot of panel c, the organisms with a lower score when using BUSCO_Augustus do not use the standard genetic code. According to the NCBI taxonomy database *Paramecium tetraurelia* (GCA_000165425.1), *Tetrahymena thermophila* (GCA_000189635.1), *Ichthyophthirius multifiliis* (GCA_000220395.1), *Stylonychia lemnae* (GCA_000751175.1) and *Halteria grandinella* (GCA_006369765.1) use the translation table number 6. When passing the correct genetic code through the “--augustus_parameters” option, Augustus is able to predict the gene models and the scores become congruent with those of BUSCO_MetaEuk. It is useful to highlight the different behaviour of the two gene predictors with respect to genomes using this alternative genetic code, as this can make a difference especially when analysing (meta)genomes of unknown organisms for which the genetic code is unknown a priori.

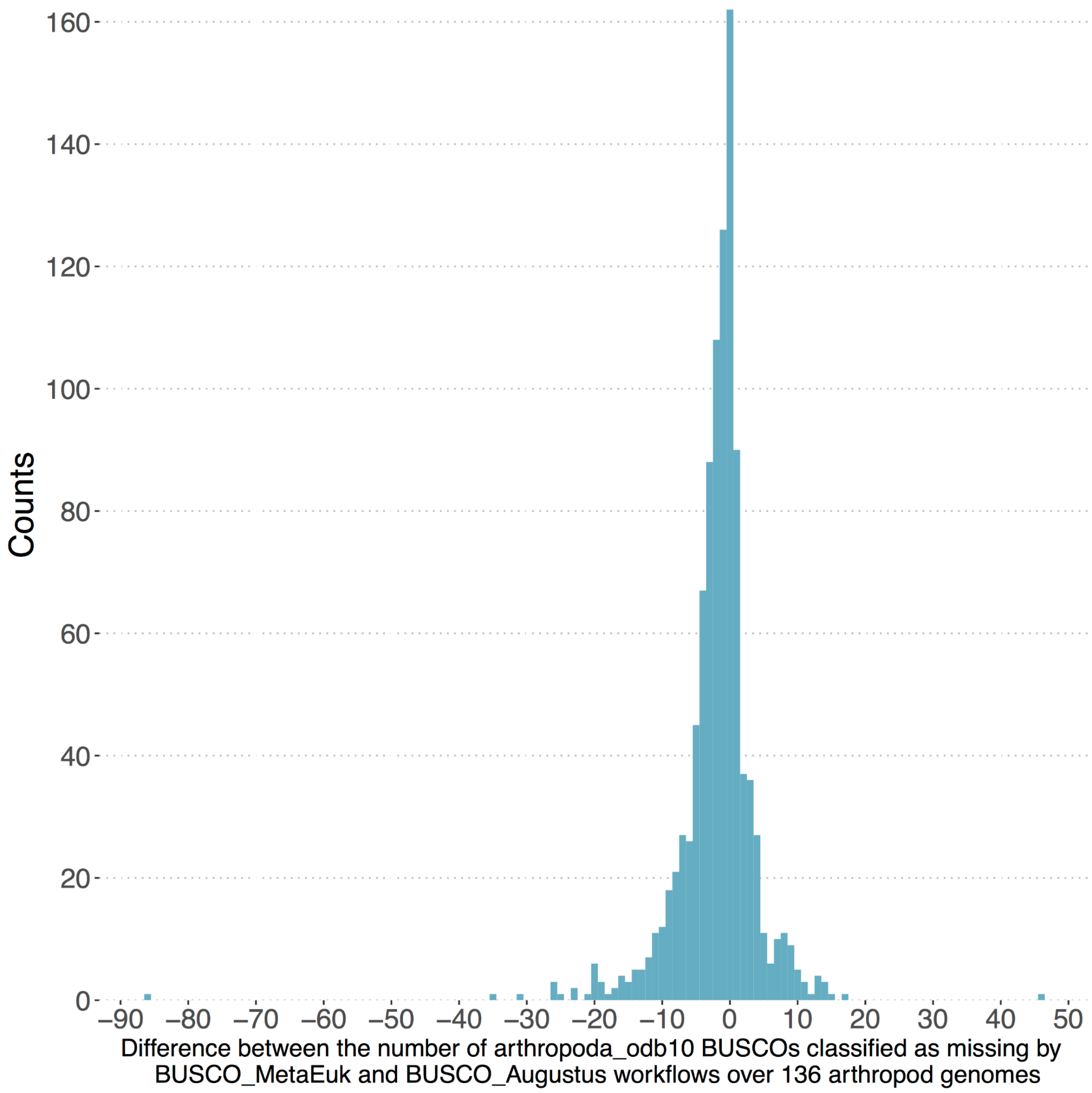

Supplementary Fig. 4. Differences in predictions of the two genome assessment workflows on a set of 139 arthropod genomes using the arthropoda_odb10 dataset (1’013 markers). x-axis represents the difference in the number of species in which each BUSCO marker was classified as missing between the two workflows, i.e. for each BUSCO marker *i*, $delta_i$ is equal to the number of genomes in which *i* is classified as missing by BUSCO_MetaEuk workflow minus the number of genomes in which *i* is classified as missing by BUSCO_Augustus workflow.

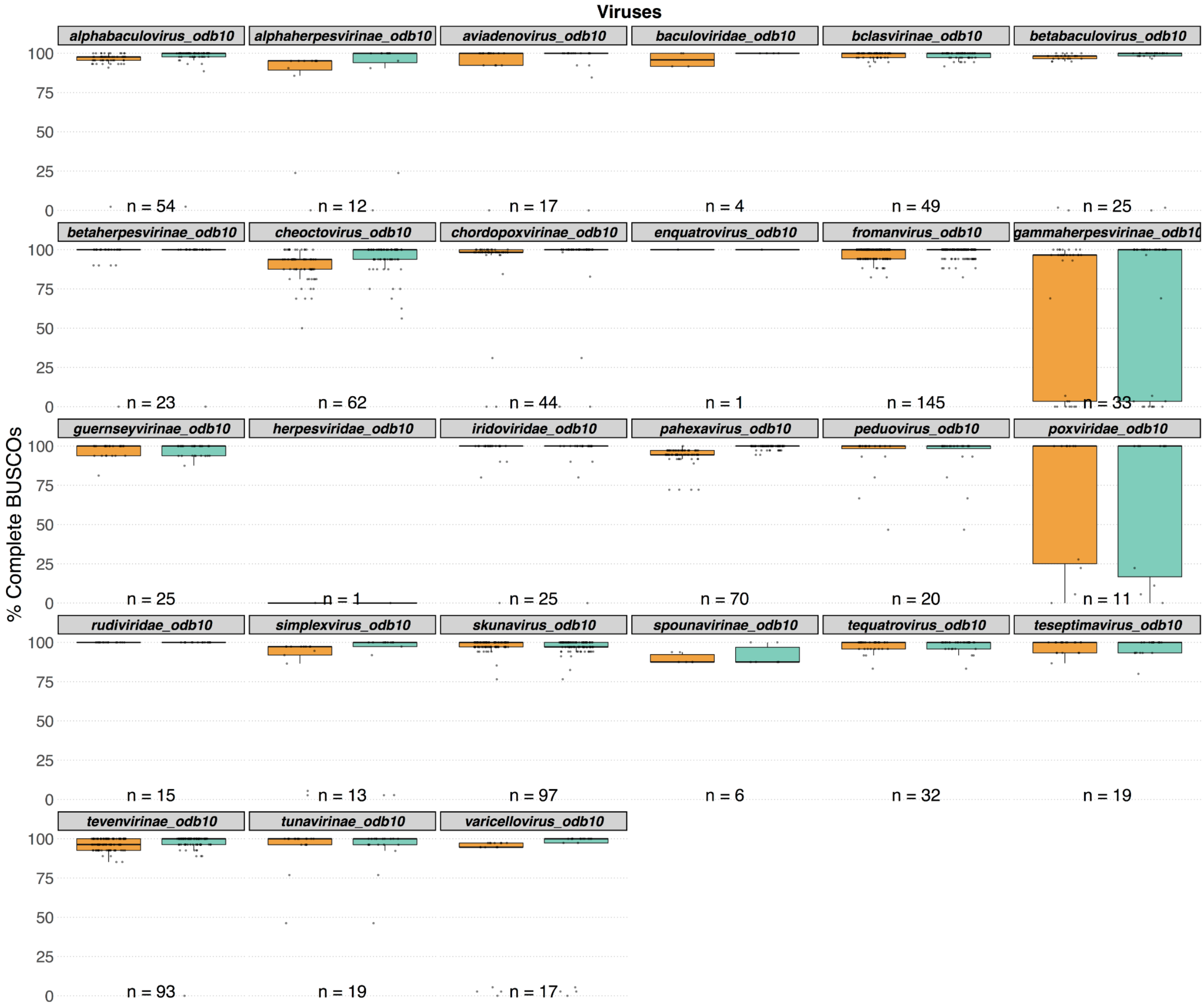

Supplementary Fig. 5. Evaluation of a set of viral genomes (orange) and corresponding gene sets (green) with the newly introduced viral BUSCO datasets (displayed at the top of each sub-panel). Viruses were sampled from the NCBI RefSeq database. In the boxplot of “gammaherpesvirinae_ob10” and “poxviridae_odb10” the genomes and gene sets that miss all or almost all BUSCO markers appear to be only small fragments of viral genomes. e.g. assembly GCF_002814975.1 (Ateline gammaherpesvirus 2) is 2.5 Kbp in length, while a common size for gammaherpesvirus is ~170 Kbp.

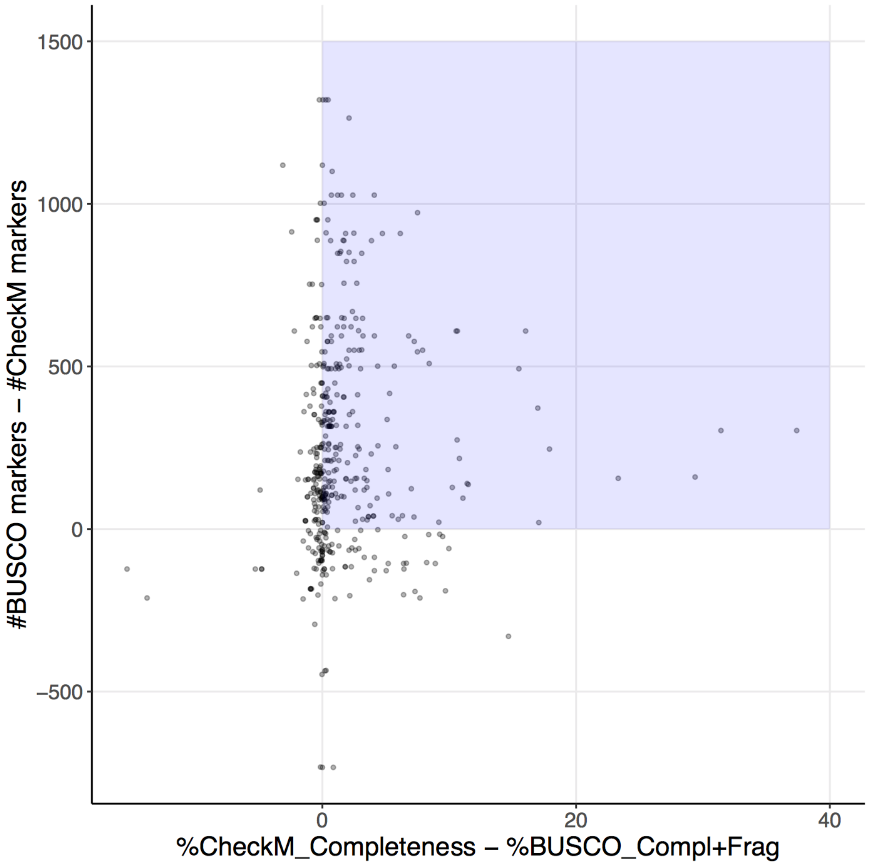

Supplementary Fig. 6. Relationship between the difference in the CheckM/BUSCO scores (x-axis) and difference in the number of markers used to assess the genomes (y-axis).

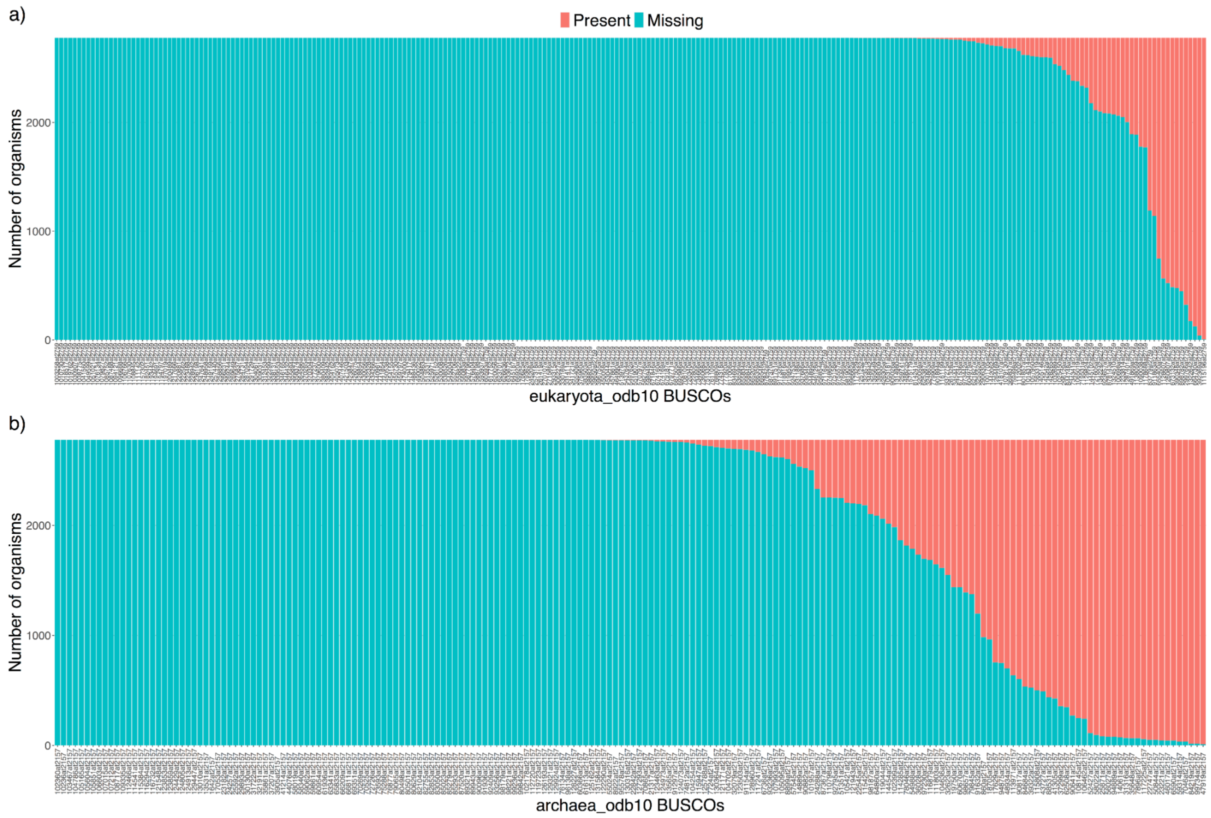

Supplementary Fig. 7. Crossmatches (red) of BUSCOs from the a) eukaryota_odb10 and b) archaea_odb10 datasets in 2’779 RefSeq bacterial genomes.

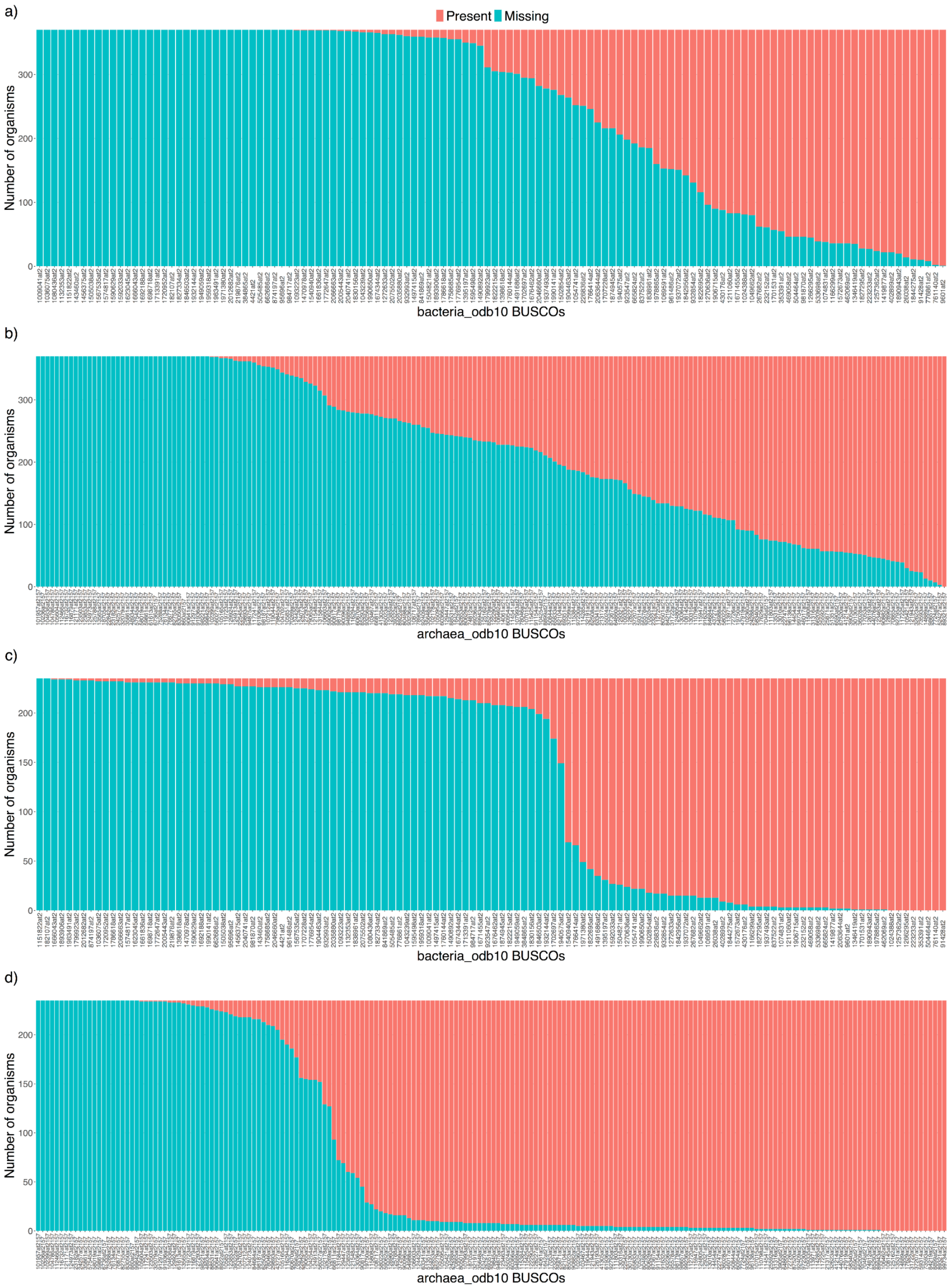

Supplementary Fig. 8. Crossmatches (red) of BUSCOs from (a and b) bacteria_odb10 and archaea_odb10 datasets in 370 fungal genomes, and (c and d**)** 235 invertebrates gene sets from RefSeq, respectively. Fragmented BUSCOs are counted as present.

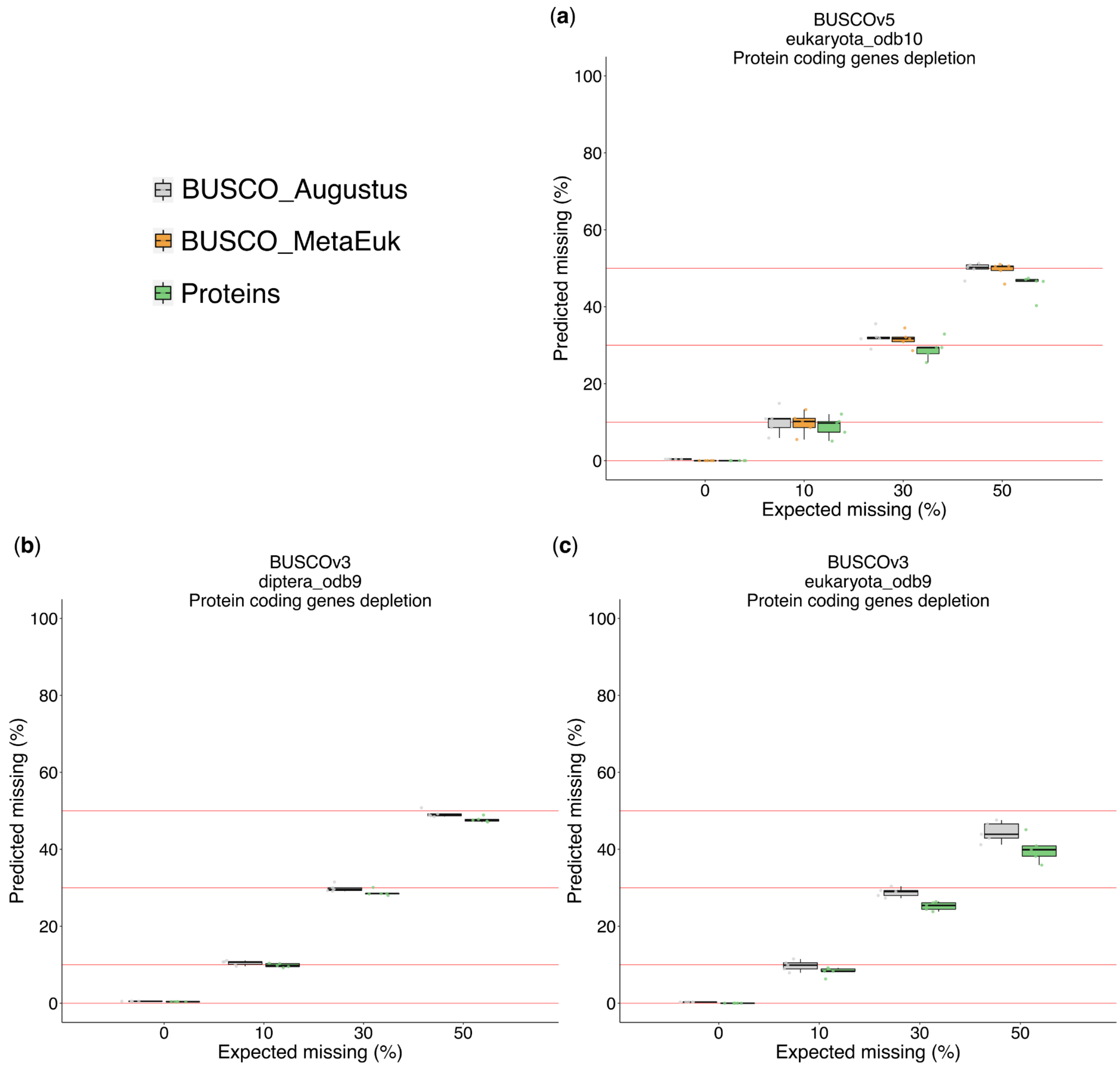

Supplementary Fig. 9. Benchmark on the genome and gene set of *Drosophila melanogaster.* Artificial depletion was made on the total genes. (a) v5 was run with the eukaryota_odb10 datasets. Figure 4a in the main text reports the benchmark using the most appropriate, i.e. specific, dataset diptera_odb10. (b and c) The same assessment using v3 with (b) dipera_odb9 and (c) eukaryota_odb9 datasets. In panels a-e, solid red lines indicate the expected missing values. Five randomly depleted versions were used for each level of depletion.

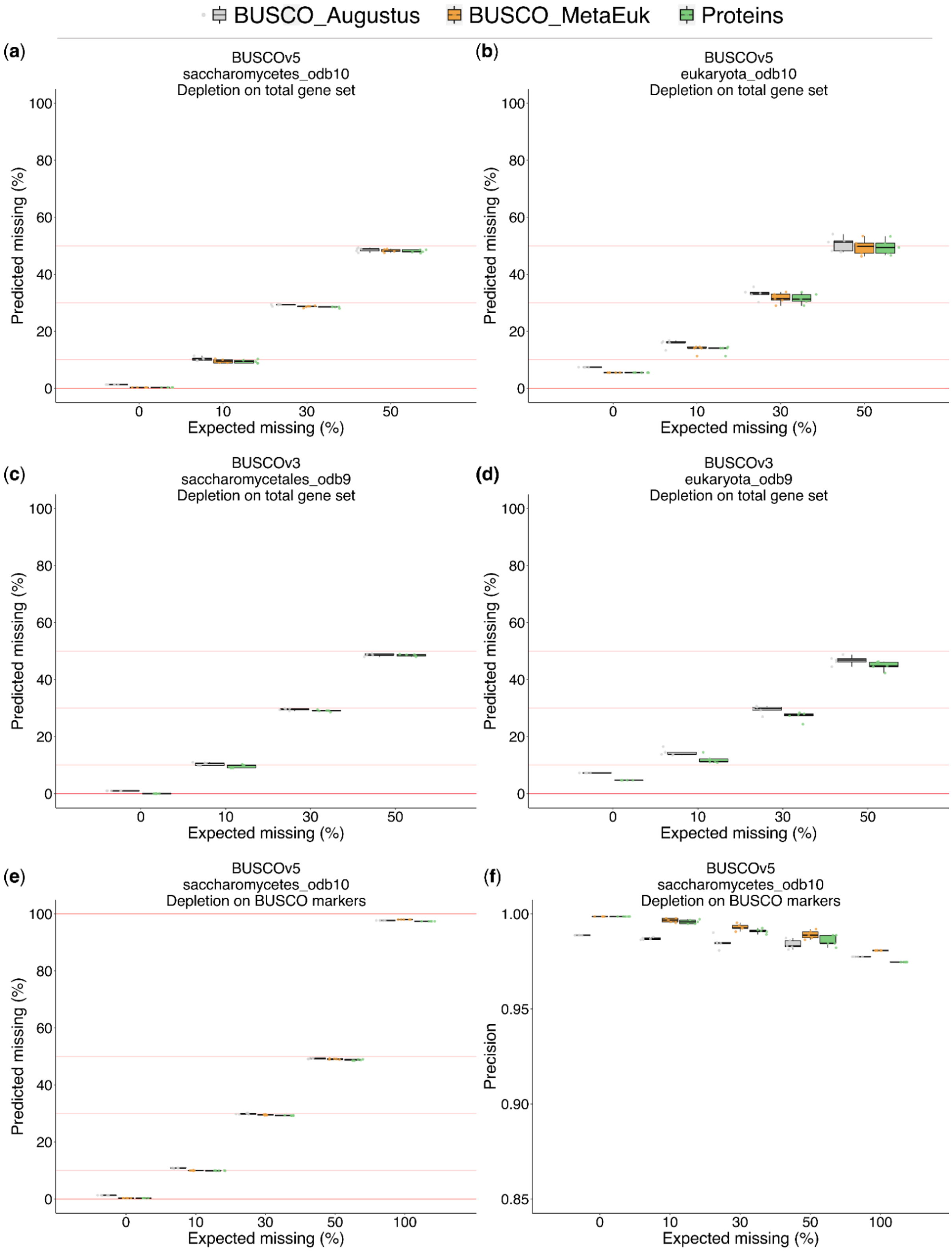

Supplementary Fig. 10. Benchmark on the genome and gene set of the yeast *Saccharomyces cerevisiae* (GCF_000146045.2). (a-d) Artificial depletion was made on the total genes and v5 was run with (a) the saccharomycetes_odb10 and (b) eukaryota_odb10 datasets; v3 was run with (c) the saccharomycetales_odb9 and (d) eukaryota_odb9 datasets. (e) Artificial depletion was exclusively made on genes matching BUSCO markers and analyzed using v5 with the most specific dataset, i.e. saccharomycetes_odb10. (f) Precisions of the predictions for the analyses of panel e. Precision is calculated taking into account genes that are present in the official gene set. In panels a-e, solid red lines indicate the expected missing values. Five randomly depleted versions were used for each level of depletion.

Supplementary tables

Note: Supplementary tables 2,4-8,10-12 are presented in the corresponding supplementary .xlsx files.

Supplementary table 1. Datasets available for BUSCO version 4 and 5 (*_odb10) along with the number of BUSCO markers and the number of species used to build the datasets. Corresponding values are shown for equivalent datasets available in version 3 (*_odb9).

BUSCO_odb10 dataset	Num of organisms	Num OGs	BUSCO_odb9 dataset	Num organisms odb9 dataset	Num OGs odb9 dataset
bacteria_odb10	4085	124	bacteria_odb9	3663	148
acidobacteria_odb10	24	601	-	-	-
actinobacteria_phylum_odb10	948	292	actinobacteria_odb9	412	352
actinobacteria_class_odb10	893	356	-	-	-
corynebacteriales_odb10	231	743	-	-	-
micrococcales_odb10	234	537	-	-	-
propionibacteriales_odb10	54	649	-	-	-
streptomycetales_odb10	145	1579	-	-	-
streptosporangiales_odb10	34	1162	-	-	-
coriobacteriia_odb10	37	422	-	-	-
coriobacteriales_odb10	25	548	-	-	-
aquificae_odb10	17	549	-	-	-
bacteroidetes-chlorobi_group_odb10	504	377	-	-	-
bacteroidetes_odb10	487	402	bacteroidetes_odb9	252	444
bacteroidia_odb10	142	538	-	-	-
bacteroidales_odb10	132	541	-	-	-
cytophagia_odb10	82	768	-	-	-
cytophagales_odb10	82	768	-	-	-
flavobacteriia_odb10	215	727	-	-	-
flavobacteriales_odb10	213	733	-	-	-
sphingobacteriia_odb10	26	1068	-	-	-
chlorobi_odb10	14	1160	-	-	-
chlamydiae_odb10	20	533	-	-	-
chloroflexi_odb10	24	271	-	-	-
cyanobacteria_odb10	141	773	cyanobacteria_odb9	112	834
chroococcales_odb10	12	1493	-	-	-
nostocales_odb10	32	1899	-	-	-

oscillatoriales_odb10	23	1493	-	-	-
synechococcales_odb10	62	788	-	-	-
firmicutes_odb10	1212	218	firmicutes_odb9	951	232
bacilli_odb10	716	302	-	-	-
bacillales_odb10	412	450	bacillales_odb9	238	526
lactobacillales_odb10	304	402	lactobacillales_odb9	330	443
clostridia_odb10	385	247	clostridia_odb9	289	254
clostridiales_odb10	347	264	-	-	-
thermoanaerobacterales_odb10	27	476	-	-	-
selenomonadales_odb10	23	796	-	-	-
tissierellia_odb10	37	379	-	-	-
tissierellales_odb10	32	433	-	-	-
fusobacteria_odb10	23	510	-	-	-
fusobacteriales_odb10	23	510	-	-	-
planctomycetes_odb10	21	571	-	-	-
proteobacteria_odb10	2337	219	proteobacteria_odb9	1520	221
alphaproteobacteria_odb10	746	432	-	-	-
rhizobiales_odb10	264	639	rhizobiales_odb9	154	686
rhizobium-agrobacterium_group_odb10	43	1937	-	-	-
rhodobacterales_odb10	180	833	-	-	-
rhodospirillales_odb10	82	617	-	-	-
rickettsiales_odb10	34	364	-	-	-
sphingomonadales_odb10	124	1018	-	-	-
betaproteobacteria_odb10	364	569	betaproteobacteria_odb9	215	582
burkholderiales_odb10	249	688	-	-	-
neisseriales_odb10	46	804	-	-	-
nitrosomonadales_odb10	36	738	-	-	-
delta-epsilon-subdivisions_odb10	233	243	deltaepsilonsub_odb9	218	296
deltaproteobacteria_odb10	124	259	-	-	-
desulfobacterales_odb10	22	599	-	-	-
desulfovibrionales_odb10	43	777	-	-	-
desulfuromonadales_odb10	22	870	-	-	-
epsilonproteobacteria_odb10	109	591	-	-	-
campylobacterales_odb10	102	628	-	-	-
gammaproteobacteria_odb10	976	366	gammaproteobacteria_odb9	721	452
alteromonadales_odb10	115	820	-	-	-

cellvibrionales_odb10	34	913	-	-	-
chromatiales_odb10	46	652	-	-	-
enterobacterales_odb10	212	440	enterobacteriales_odb9	216	781
legionellales_odb10	43	772	-	-	-
oceanospirillales_odb10	78	619	-	-	-
pasteurellales_odb10	25	1100	-	-	-
pseudomonadales_odb10	159	782	-	-	-
thiotrichales_odb10	44	491	-	-	-
vibrionales_odb10	57	1445	-	-	-
xanthomonadales_odb10	80	1152	-	-	-
spirochaetes_odb10	68	239	spirochaetes_odb9	102	237
spirochaetia_odb10	68	239	-	-	-
spirochaetales_odb10	45	345	-	-	-
synergistetes_odb10	15	655	-	-	-
tenericutes_odb10	124	150	tenericutes_odb9	68	166
mollicutes_odb10	123	151	-	-	-
entomoplasmatales_odb10	25	332	-	-	-
mycoplasmatales_odb10	79	174	-	-	-
thermotogae_odb10	23	713	-	-	-
verrucomicrobia_odb10	20	471	-	-	-
archaea_odb10	404	194	-	-	-
thaumarchaeota_odb10	17	722	-	-	-
thermoprotei_odb10	41	296	-	-	-
thermoproteales_odb10	13	404	-	-	-
sulfolobales_odb10	13	1244	-	-	-
desulfurococcales_odb10	13	491	-	-	-
euryarchaeota_odb10	344	234	-	-	-
thermoplasmata_odb10	13	340	-	-	-
methanococcales_odb10	18	958	-	-	-
methanobacteria_odb10	35	782	-	-	-
methanomicrobia_odb10	62	588	-	-	-
methanomicrobiales_odb10	21	882	-	-	-
halobacteria_odb10	165	853	-	-	-
halobacteriales_odb10	45	904	-	-	-
natrialbales_odb10	49	1368	-	-	-
haloferacales_odb10	71	1039	-	-	-
eukaryota_odb10	70	255	eukaryota_odb9	65	303

alveolata_odb10	65	171	alveolata_stramenophiles_ensembl	24	234
apicomplexa_odb10	54	446	-	-	-
aconoidasida_odb10	33	1135	-	-	-
plasmodium_odb10	23	3642	-	-	-
coccidia_odb10	20	502	-	-	-
euglenozoa_odb10	31	130	-	-	-
fungi_odb10	549	758	fungi_odb9	85	290
ascomycota_odb10	365	1706	ascomycota_odb9	75	1315
dothideomycetes_odb10	45	3786	-	-	-
capnodiales_odb10	13	3578	-	-	-
pleosporales_odb10	17	6641	-	-	-
eurotiomycetes_odb10	103	3546	eurotiomycetes_odb9	25	4046
chaetothyriales_odb10	20	6265	-	-	-
eurotiales_odb10	60	4191	-	-	-
onygenales_odb10	21	4862	-	-	-
leotiomycetes_odb10	29	3234	-	-	-
helotiales_odb10	14	5177	-	-	-
saccharomycetes_odb10	76	2137	saccharomyceta_odb9	70	1759
sordariomycetes_odb10	97	3817	sordariomyceta_odb9	30	3725
glomerellales_odb10	14	6841	-	-	-
hypocreales_odb10	50	4494	-	-	-
basidiomycota_odb10	133	1764	basidiomycota_odb9	25	1335
agaricomycetes_odb10	77	2898	-	-	-
agaricales_odb10	25	3870	-	-	-
boletales_odb10	11	4878	-	-	-
polyporales_odb10	17	4464	-	-	-
tremellomycetes_odb10	18	4284	-	-	-
microsporidia_odb10	13	600	microsporidia_odb9	14	518
mucoromycota_odb10	21	1614	-	-	-
mucorales_odb10	15	2449	-	-	-
metazoa_odb10	65	954	metazoa_odb9	65	978
arthropoda_odb10	90	1013	arthropoda_odb9	60	1066
arachnida_odb10	10	2934	-	-	-
insecta_odb10	75	1367	insecta_odb9	42	1658
endopterygota_odb10	56	2124	endopterygota_odb9	35	2442
diptera_odb10	56	3285	diptera_odb9	25	2799
hymenoptera_odb10	40	5991	hymenoptera_odb9	25	4415

lepidoptera_odb10	16	5286	-	-	-
hemiptera_odb10	16	2510	-	-	-
mollusca_odb10	7	5295	-	-	-
nematoda_odb10	7	3131	nematoda_odb9	8	982
vertebrata_odb10	67	3354	vertebrata_odb9	65	2586
actinopterygii_odb10	26	3640	actinopterygii_odb9	20	4584
cyprinodontiformes_odb10	10	15213	-	-	-
tetrapoda_odb10	38	5310	tetrapoda_odb9	55	3950
mammalia_odb10	24	9226	mammalia_odb9	50	4104
eutheria_odb10	107	11366	-	-	-
euarchontoglires_odb10	49	12692	euarchontoglires_odb9	25	6192
glires_odb10	22	13798	-	-	-
primates_odb10	25	13780	-	-	-
laurasiatheria_odb10	51	12234	laurasiatheria_odb9	25	6253
carnivora_odb10	12	14502	-	-	-
cetartiodactyla_odb10	21	13335	-	-	-
sauropsida_odb10	76	7480	-	-	-
aves_odb10	62	8340	aves_odb9	40	4915
passeriformes_odb10	15	10844	-	-	-
stramenopiles_odb10	27	100	-	-	-
viridiplantae_odb10	57	425	-	-	-
chlorophyta_odb10	16	1519	-	-	-
embryophyta_odb10	50	1614	embryophyta_odb9	20	1440
liliopsida_odb10	15	3236	-	-	-
poales_odb10	12	4896	-	-	-
eudicots_odb10	31	2326	-	-	-
brassicales_odb10	10	4596	-	-	-
fabales_odb10	10	5366	-	-	-
solanales_odb10	11	5950	-	-	-
alphabaculovirus_odb10	43	44	-	-	-
alphaherpesvirinae_odb10	31	21	-	-	-
aviadenovirus_odb10	12	13	-	-	-
baculoviridae_odb10	70	12	-	-	-
bclasvirinae_odb10	13	36	-	-	-
betabaculovirus_odb10	21	58	-	-	-
betaherpesvirinae_odb10	21	10	-	-	-
cheoctovirus_odb10	11	16	-	-	-
chordopoxvirinae_odb10	30	58	-	-	-

enquatrovirus_odb10	14	12	-	-	-
fromanvirus_odb10	10	17	-	-	-
gammaherpesvirinae_odb10	19	29	-	-	-
guernseyvirinae_odb10	11	16	-	-	-
herpesviridae_odb10	71	12	-	-	-
iridoviridae_odb10	17	10	-	-	-
pahexavirus_odb10	30	36	-	-	-
peduovirus_odb10	16	15	-	-	-
poxviridae_odb10	39	18	-	-	-
rudiviridae_odb10	11	11	-	-	-
simplexvirus_odb10	11	37	-	-	-
skunavirus_odb10	15	34	-	-	-
spounavirinae_odb10	12	16	-	-	-
tequatrovirus_odb10	30	24	-	-	-
teseptimavirus_odb10	23	15	-	-	-
tevenvirinae_odb10	51	27	-	-	-
tunavirinae_odb10	13	26	-	-	-
varicellovirus_odb10	10	37	-	-	-

Supplementary table 3. Breakdown of the main differences among BUSCO v3, v4 and v5.

	**v3**	**v4**	**v5**
**Protein mode***	Available	Available	Available
**Genome mode BUSCO_Augustus workflow**	Available for eukaryotes and prokaryotes	Available for eukaryotes	Optional for eukaryotes
**Genome mode BUSCO_Prodigal workflow**	X	Available for prokaryotes	Available for prokaryotes and viruses
**Genome mode BUSCO_MetaEuk workflow**	X	X	Available for eukaryotes
**Transcriptome mode**	Available (using BLAST)	Available (using BLAST)	Available (using BLAST for prokaryotes; using MetaEuk for eukaryotes)
**Auto-lineage workflow**	X	Available for genome, protein and transcriptome modes for both eukaryotes and prokaryotes	Available for genome, protein and transcriptome modes for eukaryotes, prokaryotes and viruses
**Virus pipeline (subset of clades)**	X	X	Available
**Batch mode**	X	X	Available
**Automatic dataset download**	X	Available	Available
**Config file**	Required	Required	Optional
**Dataset version**	odb9	odb10	odb10
**# available datasets**	49	166	193**

* The protein mode always depends on HMMER and has not changed.
** 27 viral datasets were added.

Supplementary table 9. Comparison of the computational resources needed to analyze 436 prokaryotic genomes with BUSCO (batch mode + “--auto-lineage-prok”), CheckM (lineage_wf) and a Snakemake/BUSCO workflow.

	Num cpus	Total runtime for 436 genomes (min)	Max mem (Gb)	Mean time per genome (sec)
**BUSCO**	30	257	11.2	35
	12	304	9.8	42
	8	340	10.3	48
**CheckM**	30	96	70.2	13
	12	131	70.2	18
	8	161	70.2	22
**BUSCO with Snakemake**	30 (5 per task)	95	11.2	13
